# Assessing satisfaction of search in virtual mammograms for experienced and novice searchers

**DOI:** 10.1117/1.JMI.10.S1.S11917

**Published:** 2023-07-21

**Authors:** Stephen H. Adamo, Nelson Roque, Bruno Barufaldi, Joseph Schmidt, Claudia Mello-Thoms, Miguel Lago

**Affiliations:** a University of Central Florida, Orlando, Florida, United States; b University of Pennsylvania, Philadelphia, Pennsylvania, United States; c University of Iowa, Iowa City, Iowa, United States; d U.S. Food and Drug Administration, Silver Spring, Maryland, United States

**Keywords:** satisfaction of search, subsequent search misses, visual search, attentional template, breast imaging

## Abstract

**Purpose:**

Satisfaction of search (SOS) is a phenomenon where searchers are more likely to miss a lesion/target after detecting a first lesion/target. Here, we investigated SOS for masses and calcifications in virtual mammograms with experienced and novice searchers to determine the extent to which: (1) SOS affects breast lesion detection, (2) similarity between lesions impacts detection, and (3) experience impacts SOS rates.

**Approach:**

The open virtual clinical trials framework was used to simulate the breast anatomy of patients, and up to two simulated masses and/or single-calcifications were inserted into the breast models. Experienced searchers (residents, fellows, and radiologists with breast imaging experience) and novice searchers (undergraduates who had no breast imaging experience) were instructed to search for up to two lesions (masses and calcifications) per image.

**Results:**

2×2 mixed factors analysis of variances (ANOVAs) were run with: (1) single versus second lesion hit rates, (2) similar versus dissimilar second-lesion hit rates, and (3) similar versus dissimilar second-lesion response times as within-subject factors and experience as the between subject’s factor. The ANOVAs demonstrated that: (1) experienced and novice searchers made a significant amount of SOS errors, (2) similarity had little impact on experienced searchers, but novice searchers were more likely to miss a dissimilar second lesion compared to when it was similar to a detected first lesion, (3) experienced and novice searchers were faster at finding similar compared to dissimilar second lesions.

**Conclusions:**

We demonstrated that SOS is a significant cause of lesion misses in virtual mammograms and that reader experience impacts detection rates for similar compared to dissimilar abnormalities. These results suggest that experience may impact strategy and/or recognition with theoretical implications for determining why SOS occurs.

## 1 Introduction

**Fig. 1:**
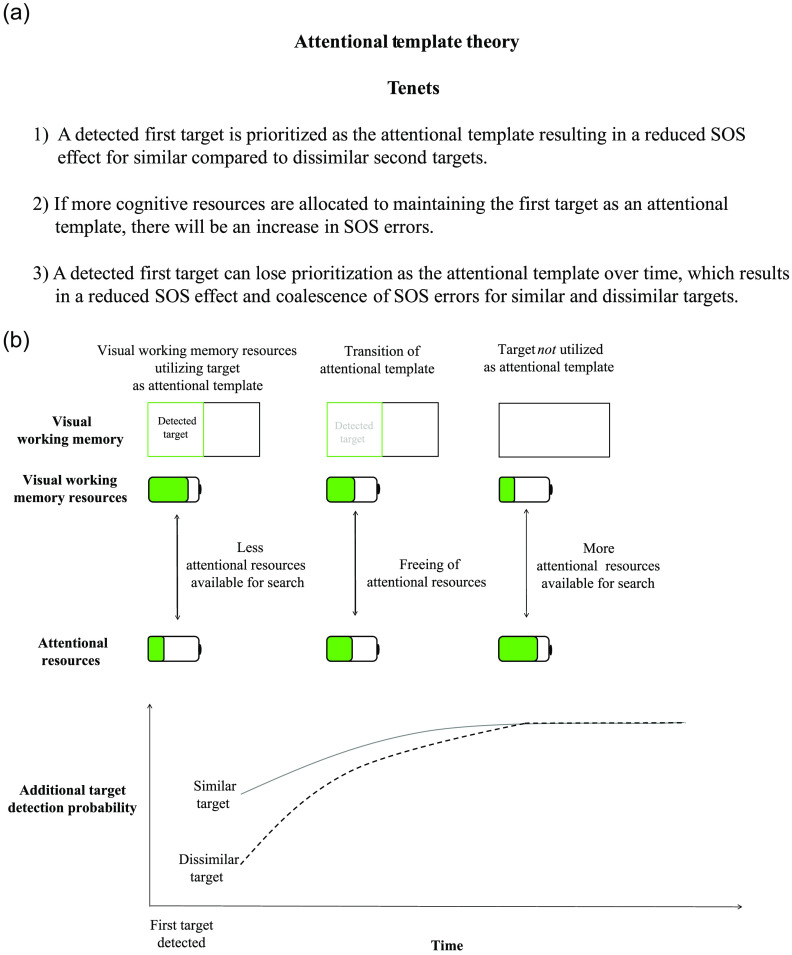
(Previously published in Ref. 17): (a) attentional template theory tenets. An attentional template is known in cognitive science as an active representation of the search goal held within visual working memory. The attentional template theory predicts that the maintenance of a detected target as a searchers’ attentional template is why SOS errors occur. The tenets of the attentional template theory were motivated by the SOS error literature and the broader visual working memory, attention, and visual search literature. (b) Time course of the fluctuation of working memory and attentional resources in a multiple target search. The battery illustration depicts the capacity limitations of visual working memory and attention. The double-sided arrows between the batteries illustrate the flow of a shared cognitive resource that underlies both visual working memory and attention, and that when one cognitive process is prioritized, the remaining cognitive process is hindered. After first target detection, visual working memory resources are prioritized to maintain the first target as an attentional template. Consequently, fewer attentional resources are available, which will decrease the probability of additional target detection. After detecting a first target, the detection of similar targets will improve compared to dissimilar targets because a first target attentional template will prime attention toward similar targets. Furthermore, the detection of similar, second targets will be faster than dissimilar second targets. Over time the first target may lose its prioritization as the attentional template, which will free up visual working memory resulting in improved and unbiased detection for similar and dissimilar targets.

Satisfaction of search (SOS) is a known problem within cognitive science and radiology where searchers are more likely to miss a target/lesion after detecting a first target/lesion.^1^^–^^3^ While initially discovered in radiographs and medical images,^3^^–^^5^ cognitive science researchers, researchers who study human cognition and topics, such as attention, memory, and perception, have investigated SOS by replicating and confirming the existence of SOS with novice searchers (i.e., college students) in simplified search displays. This allows for the evaluation of a variety of search conditions such as target shape, color, target location, etc.^1^ Also known as “subsequent search misses” in cognitive science, cognitive researchers primarily test the theorized causes of SOS—satisfaction,^2^^,^^3^ perceptual set,^4^ and resource depletion.^5^ The satisfaction theory, and the origin of the name “SOS,” hypothesizes that searchers become “satisfied” with the image’s meaning after finding a target, causing searchers to prematurely terminate their search and miss an additional target. In addition, the perceptual set theory predicts that the detection of one target primes searchers to find similar targets compared to dissimilar targets making it more likely to miss a dissimilar second target. Finally, the resource depletion theory suggests that cognitive resources are allocated to the processing of a first target, leaving them less readily available to detect an additional target. While cognitive science has demonstrated support for the three theories: satisfaction,^6^^–^^8^ perceptual set,^9^^–^^11^ and resource depletion,^12^^–^^16^ the theories remained disparate, with no mechanistic explanation for their predictions. More recently, the attentional template theory has been proposed,^17^ which bridges the findings in support of each theory for a more unified, mechanistic explanation for SOS errors (See Fig. 1).

The attentional template theory provides a mechanistic account of SOS errors. It predicts that a detected first target becomes an observer’s “attentional template,” which increases the likelihood of missing a different additional target. In cognitive science, an attentional template is known as an active representation of the search goal held within visual working memory (e.g., searchers are shown a red circle beforehand and then search for a red circle in a search display).^18^ When searchers are shown a cue (i.e., a potential target) before search, response times (a common way to assess the effectiveness of attentional template/ attention in visual search in cognitive science^19^) are faster and hit rates are higher when targets have cue similar features relative to cue dissimilar features because attention is guided towards similar targets.^20^^,^^21^

Three key findings from the cognitive science literature suggest that the attentional template is the mechanistic explanation for the SOS theories. Satisfaction theory—searchers are less likely to maintain a first target as an attentional template as more time passes between seeing the template and finding the target.^18^^,^^20^^,^^21^ This finding explains why searchers in simplified search images are prone to SOS when searching for less time after finding a first target.^6^ Perceptual set theory—searchers are attentionally biased to search for targets perceptually similar to the template.^22^^–^^24^ This finding explains why searchers are prone to SOS when a second target is dissimilar to a detected target.^9^^–^^11^ Resource depletion theory—attentional templates are thought to be maintained within visual working memory, resulting in the consumption of visual working memory and attentional resources.^25^^,^^26^ This finding explains why searchers make fewer SOS errors when visual working memory and attentional resources are readily available.^12^^–^^15^

Similar to cognitive science, early research radiology attempted to identify why SOS errors occurred yielding the satisfaction theory,^2^^,^^3^ perceptual set theory,^4^ and resource depletion theory.^5^ However, medical image perception researchers have primarily focused on identifying the pervasiveness of SOS across different types of medical images, including chest radiography, abdominal radiography, skeletal radiography, and multiple-trauma patient scans.^27^^–^^33^

Surprisingly, while SOS has been examined across many different types of medical images, SOS has not been studied in breast cancer detection. Ten to 30% of breast cancers per year are not reported in mammography screenings^34^ and in the United States, breast cancer accounts for 31% of all new cancer diagnoses in women.^35^ The extent to which SOS affects breast cancer is critical because it may provide insight into why some breast cancers are missed.

Our goal was to investigate SOS in breast lesion detection and test one of the key predictions of the attentional template theory—does the detection of a specific lesion (e.g., a mass) become a searcher’s attentional template and impact detection for an additional similar lesion (e.g., another mass) compared to a different lesion (e.g., a calcification) in X-ray images of simulated breast anatomies? In other words, (1) are searchers are more likely to miss dissimilar second lesions compared to similar second lesions; and (2) if they do find a second lesion, are they faster at finding similar second lesions compared to dissimilar second lesions? Furthermore, we investigated whether experience impacts the SOS effect. If there are differences in SOS errors between searchers who are experienced in reading medical images and searchers who are not, this could ultimately provide insight into medical education and training.

## 2 Methods

### 2.1 Images

**Fig. 2:**
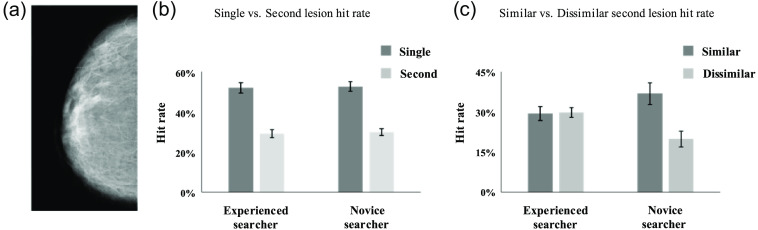
(a) Sample virtual breast mammogram. (b) Graph of single and second lesion hit rate (i.e., to determine whether there was an SOS effect). (c) Graph of second lesion hit rates when they were similar and dissimilar to a detected first lesion (i.e., to determine whether there was a similarity effect).

The open virtual clinical trials (VCTs) framework was used to generate virtual images of the breast anatomy [See Fig. 2(a)], and the images created by Open VCT have been used in prior research to investigate how radiologists search for breast lesions.^36^ The central slices of a digital breast tomogram (i.e., a segmented-3D representation of breast tissue) were used as the images for the task to simulate a 2D mammogram. [Fig. 2(a); Briona, version 7.12].^37^ The breast parenchyma includes the simulation of Coopers’ ligaments, skin, adipose, and glandular tissue compartments. The virtual images were simulated using a voxel size of $0.1\;{\mathrm{mm}}^{3}$, a total volume of 700 ml, 63.3 mm mediolateral-compressed thickness, and 15% to 25% of glandular tissue.

Up to two simulated lesions were inserted into the images representing patients with two masses, two distinct calcifications (i.e., not calcification clusters), and a combination of one mass and one distinct calcification.^37^^,^^38^ The same size and shape mass and calcification models were used across images (e.g., the mass was the same size, shape, density, etc., across all images). While the word “similar” is used to describe the relationship between the relationship between a first and second target when they are from the same category (e.g., masses), the two lesions in the similar condition were identical in shape and size—a limitation further described in the Discussion section. These lesions were embedded into the images at random coordinates within the most-central plane. The lesions were inserted at random x and y coordinates, excluding locations close to the periphery of the image (10 mm of the skin and chest wall). Because the exact coordinates of the lesions were known (i.e., they were virtual breast images), Adamo and Lago excluded images where the abnormalities were completely obscured by dense tissue.

The images were also created as near-identical triads with two single-lesion images paired with every dual-lesion image. For example, if a dual-lesion image contained a mass and calcification, one matching single image contained the calcification (mass removed), and the other contained the mass (calcification removed). A total of 15 triads (45 total images) were created based on the above criteria: 15 unique images containing 2 lesions (5 dual-masses, 5 dual-calcifications, and 5 mass and calcification images) and 30 containing one lesion (15 single-mass and 15 single-calcification). An additional 7 images containing no lesions were added that were not part of the triads resulting in a total of 52 images. As our main focus was on the images with lesions, lesion-free images were primarily added so participants were willing to interpret a case as normal rather than expecting a lesion in every image. Six practice trials preceded the experimental trials and included one trial from each condition so the participants would learn which images to expect (i.e., single: mass and calcification, dual: masses, calcifications, mass and calcification, and a no lesion image). Feedback was provided and the experimenter was there to help the participants if needed during the practice trials only (e.g., if participants needed clarification about what were and were not lesions).

### 2.2 Participants and Procedures

Thirty-four experienced searchers (16 residents, 15 fellows/radiologists, and 3 no responses) with breast imaging experience were recruited at the Radiological Society of North America (RSNA) annual meeting. Of the radiologists, seven self-reported that they were breast imagers that primarily searched breast images, five reported that they were general radiologists, and three were of other disciplines that had experience with breast images but primarily focused on other modalities (but were comfortable participating in the task).

Forty-two novice searchers (i.e., naive readers without medical knowledge who had no medical imaging experience) were recruited from the University of Central Florida participant pool. Participant number was based on previous studies that compared experienced and novice searchers (∼30 participants)^39^ and extra novice searchers were recruited because we did not know if they could find any lesions at all. No participants were excluded because of poor performance.

Participants were told that there were up to two lesions per image and to perform a mouse click on what they believed was a lesion. If the click was within 25 pixels of the center of the lesion, it was considered a hit. There was no time limit for each image, but the experiment, on average, lasted 30 mins (the time limit mandated by RSNA). Images were presented with Matlab via Psychtoolbox^40^ on a Dell P2418HZ Color profile monitor with a 1080×1920 resolution vertical monitor. The images were fitted to the size of the monitor vertically and appeared on the right half of the screen [see Fig. 2(a)]. Participants could not zoom, pan, or adjust the image’s window/level settings in this experiment. The University of Central Florida Institutional Review Board approved the study. All participants provided verbal informed consent.

### 2.3 SOS Calculation

We implemented the methods and calculations, which have been previously shown to mitigate bias. These methods specifically address the potential for inflating the true measure of the SOS effect.^41^ Using matched images, this setup allows us to determine the impact a detected lesion has on a second lesion—the hit rate for the second lesion is compared to the hit rate of its single-lesion, matched image. The single-lesion image for the lesion found first in the dual-lesion image is not included in the SOS calculation. This design keeps everything that perceptually impacts detection (e.g., breast internal tissue distribution) constant, and only a first lesion’s impact on a second lesion is quantified.

If matching images are not used, the SOS calculation may likely be inflated because when there are two targets/lesions present, the target/lesion not found first by an observer must be the more “difficult” target to find for that observer. Therefore, if displays are not matched/triads, comparing the hit rate for single target/lesion images to the second target/lesion hit rate could be biased by the image’s properties (e.g., the second lesion missed may be because it is in a cluttered/crowded part of the image) rather than the direct impact that detecting a “first” target/lesion has on a second target/lesion. Virtual breast images allowed us to create three identical copies with only abnormalities added or removed. Matched images never appeared within three sequential trials to reduce potential memory effects.

## 3 Results

### 3.1 Overall SOS Rates, False Alarms Rates, and Response Times

To determine whether there was an SOS effect for virtual mammograms, a 2×2 analysis of variance (ANOVA) with lesion hit rate (single versus dual) and experience as a between-subjects factor (novice versus experienced searchers) was conducted. The main effect of single (M=52.2%) versus second lesion (M=29.6%) was significant (F(1,74)=156.0, p<0.001), with a 22.6% SOS effect [See Fig. 2(b)]. The between-subjects effect of experience (F(1,74)=0.08, p=0.77*)* and the interaction (F(1,74)=0.001, p=0.97) were not significant. For the trials used in the SOS analyses, independent sample two-tailed t-tests were also calculated for searchers’ single, dual, and overall (i.e., single and dual trials collapsed together) false positive rates (i.e., where participants clicked in a non-lesion location). There was no significant difference between novice and experienced searchers for single (novice searchers: M=55.9%, experienced searchers M=51.4%; t(74)=0.94, p=0.35), dual (novice searchers: M=41.1%, experienced searchers M=34.1%; t(74)=1.52, p=0.13), and overall false positive rates (novice searchers: M=62.5%, experienced searchers: M=56.4%; t(74)=1.43, p=0.16). When comparing response times on correct trials, experienced searchers’ response times were faster in single- (novice searchers: 25.8 s, experienced searchers: 15.9 s; t(74)=3.22, p=0.002) and dual-lesion (novice searchers: 16.7 s; experienced searchers: 12.2 s; t(73)=2.23, p=0.03) trials demonstrating they were faster to complete their search for lesions of the virtual mammograms.

### 3.2 Similar versus Dissimilar Lesions Comparisons

To determine whether observers were more accurate in detecting a second lesion that was similar to the first lesion (e.g., is a calcification more likely to be detected than a mass if a calcification is detected first), a 2×2 ANOVA with second-target hit rate (similar vs. dissimilar) and experience as a between-subjects factor (novice versus experienced searchers) was conducted. The main effect of similar (M=33.4%) versus dissimilar (M=24.3%) was significant (F(1,74)=8.91, p=0.004). The between-subjects effect of experience was not significant (F(1,74)=0.19, p=0.66), but the interaction was significant (F(1,74)=9.72, p=0.003). Follow-up t-tests were conducted to examine the locus of similarity by experience interaction. When lesions were different, experienced searchers (M=29.8%) were more accurate compared to novice searchers (M=18.9%; t(74)=2.04, p=0.045) but were less accurate when lesions were similar [experienced searchers: M=29.4%, novice searchers: M=36.7%; t(74)=2.43, p=0.018; See Fig. 2(c)]. Additional follow-up paired samples, one-tailed t-tests were used to test whether the first lesion acted as an attentional template as predicted by the attentional template theory (i.e., are similar second lesions detected more accurately than dissimilar second lesions). In this case, there were no differences between similar versus different second lesions for experienced searchers (t(33)=0.73, p=0.47). However, novice searchers were significantly less accurate in detecting different second lesions compared to similar second lesions (t(41)=5.48, p<0.001).

The difference in response times between detecting similar and dissimilar second lesions was investigated to further test whether the first lesion was acting as an attention template. For example, if a first mass was detected in 4 s and a second mass was detected in 12 s, the response time difference would be 8 s for this dual-mass, similar lesion trial. A 2×2 ANOVA with the difference in response time (similar versus dissimilar) and experience as a between-subjects factor was conducted. Participants who either: did not find a second lesion: (1) in the similar condition, (2) in the dissimilar condition, or (3) in both were filtered out (novice searchers = 13; experienced searchers = 9) because a correct detection of both lesions was needed in the similar and dissimilar conditions to conduct the response time difference analyses. There was a significant main effect for similarity (F(1,50)=5.74, p=0.02) demonstrating that less time was required between finding a first lesion and a similar second lesion compared to a dissimilar second lesion. In addition, there was a significant between-subjects effect demonstrating that experienced searchers were faster than novice searchers (F(1,50)=6.08, p=0.02), but not a significant interaction (F(1,50)=1.25, p=0.27). Additional follow-up, one-tailed t-test demonstrated that both novice searchers (similar = 5.8 s, dissimilar = 10.8 s; t(27)=2.18, p=0.037) and experienced searchers (similar = 3.9 s, dissimilar = 5.7 s; t(24)=2.69, p=0.01) took significantly less time between first and second lesion detection to find a similar lesion compared to a dissimilar lesion. This finding further supports the prediction that the first lesion is acting as an attentional template as searchers are faster to find a similar second lesion compared to a dissimilar second lesion.

### 3.3 Exploratory Analyses – Modeling Trial-level Data

We conducted an exploratory analysis to explore the potential benefits of observer experience on overall search time, leveraging mixed-effect modeling. Unlike the other analyses of this study that were restricted to only dual-lesion trials and their “matching” single-lesion trials (see 2.3 SOS calculation section), these analyses utilized the entire data set to determine learning rates from the beginning of the experiment to the end of the experiment and how novice searchers compared to experienced). This study leverages mixed-effect modeling to account for inter-observer variability (i.e., random intercept per participant), while simultaneously exploring effects influencing search time in this study (i.e., practice, number of targets, and stimuli complexity).

In a stepwise fashion, models leading up to the full model (i.e., a model containing fixed effects for trial number and whether the participant has imaging experience; See Table 1) were compared against an unconditional means model to determine if any of the fixed effects significantly improve model fit. The full model was the best performing model (i.e., a model containing fixed effects for trial number, and whether the participant was a novice or experienced searcher ($X^{2}$ (2, N=76) = 14.961, p<0.001).

**Table 1:** Table of the exploratory mixed effect modeling results.

	Total time searching		
Predictors	Estimates	CI	p
(Intercept)	32.87	28.76 to 36.97	<0.001
Has experience	−11.28	−17.19 to −5.37	<0.001
Global trial	−0.16	−0.20 to −0.12	<0.001
Number of targets	−2.62	−3.39 to −1.85	<0.001
False alarm	2.33	1.57 to 3.08	<0.001
Experienced * global trial	0.08	0.01 to 0.14	0.023
Random effects			
$\sigma^{2}$	235.75		
$\tau_{00\;\text{subject id}}$	151.89		
ICC	0.39		
$N_{\text{subject id}}$	76		
Observations	3915		
Marginal $R^{2}/\text{Conditional}\;R^{2}$	0.078/0.439		

**Fig. 3:**
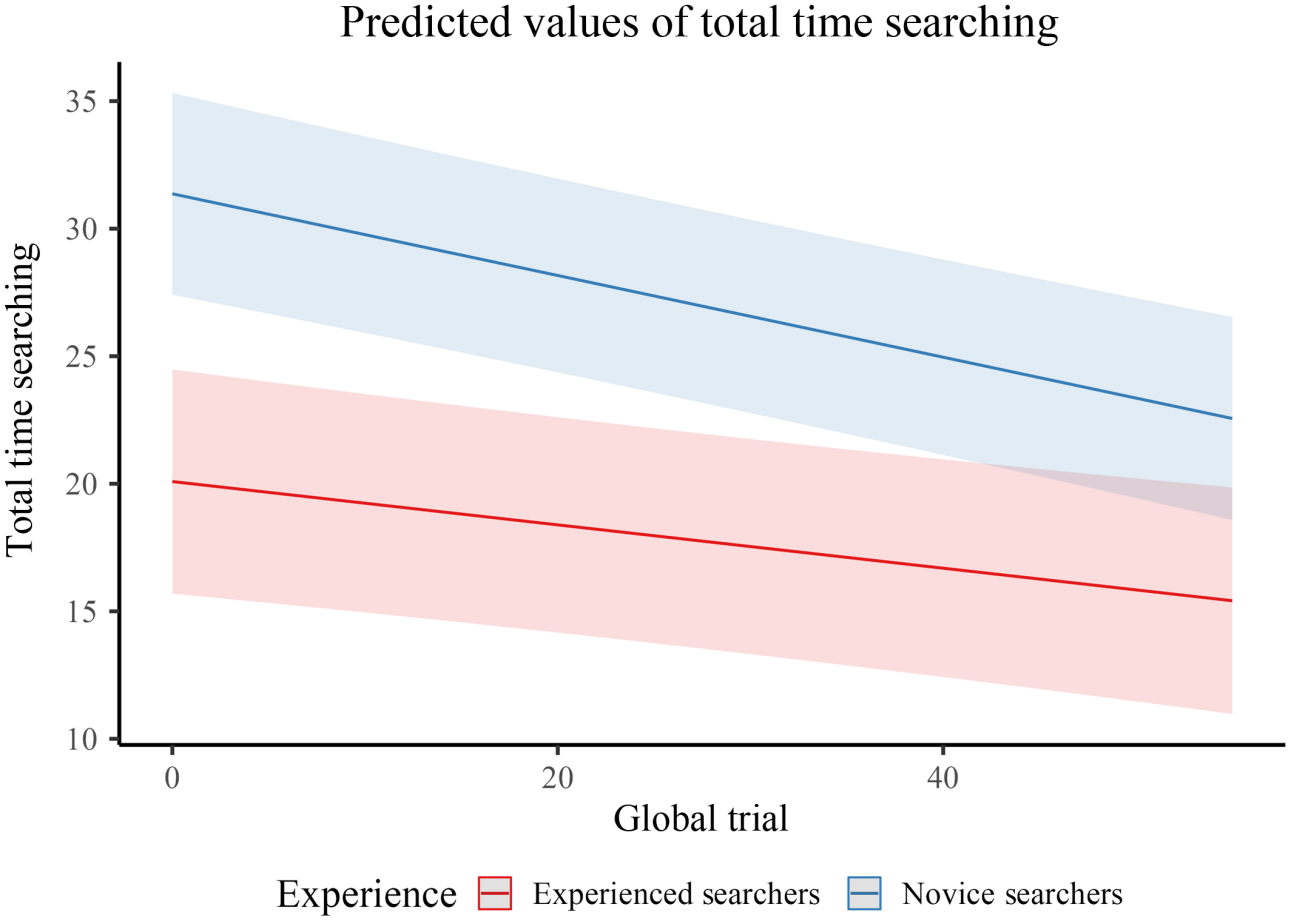
Predicted values of total time searching from the full model. The “red” color represents the 1st trial and the “blue” color represents the last trial (i.e., trial 52). The shaded color around the lines represents 95% confidence intervals. Notice the difference in the mean value for trial 1 compared to trial 52 for novice searchers compared with experienced searchers—novice searchers have a steeper learning curve, as would be predicted given that this was their first time searching breast images.

First, a substantial amount of variation in trial response time (i.e., how long observers searched for on each trial regardless of whether a target was found or not) is explained by inter-observer differences (i.e., marginal $R^{2}$ (i.e., fixed effects only) = 0.078; conditional $R^{2}$ (i.e., fixed and random effects) = 0.439; and intraclasscorrections(×ICC)=0.39). The fixed effects alone do not assume much of the variance (∼8%) in the model; adding participant intercepts assumed ∼36% of the variance. Participants responded significantly more quickly for each trial (β=−0.16, p<0.001), for each unit increase in the number of lesions present per trial (β=−2.62, p<0.001), and if they were a novice or an experienced searcher (β=−11.28, p<0.001). However, searchers were slower if they false alarmed (β=2.33, p<0.001). There was also a significant interaction (β=0.07, p=0.023), such that from the beginning of the experiment (trial 1) to the end (trial 52), the gap between experienced and novice searchers was greater in the initial period and shrunk over time. In other words, while novice and experienced searchers searched faster throughout the experiment, novice searchers took longer to search at the experiment’s beginning (and end) than experienced searchers, and the learning curve was steeper for novice searchers; See Fig. 3).

## 4 Discussion

Here, we established that the SOS effect (an increased likelihood of missing a lesion after a first lesion is detected) is a viable cause of misses in virtual breast images. Novice searchers (university undergraduates with no radiological experience) and experienced searchers (residents, fellows, and radiologists with breast imaging experience) searched for up to two masses and/or calcifications in virtual mammograms [i.e., single-slice digital breast tomosynthesis (DBTs)]. Overall, SOS rates did not differ between novice and experienced searchers but differed in how their SOS rates manifested. Novice searchers were more likely to make SOS errors when lesions differed (e.g., a mass and calcification were within the virtual mammogram), which supports the most recent SOS theory—the attentional template theory. Experienced searchers were just as likely to make SOS errors regardless of whether the lesions differed. Experienced searchers were also faster at finding lesions.

Regarding the exploratory analyses, novice searchers showed a greater learning rate and were slower throughout the experiment. These analyses further emphasize that experience matters – in terms of individual differences and within the experiment—and these distinct but related sources of variation should be carefully considered when comparing novice to experienced searchers. Overall, our findings have direct implications for breast cancer detection, speak to SOS theory, and the role of experience.

### 4.1 SOS Theory

The attentional template theory predicts that, after searchers find a first lesion, it becomes their attentional template. Consequently, they: (1) are more likely overall to miss an additional lesion as cognitive resources (i.e., attention and visual working memory) are allocated to maintaining the first target as an attentional template; (2) have SOS errors at disproportionate rates for similar and dissimilar lesions (because an attentional template biases searchers towards lesions similar to the template), and (3) are more likely to make an SOS error the less time they search after finding a first target and will require more time to find dissimilar compared to similar targets.

While not all the predictions of the attentional template were explicitly tested here (e.g., the study was not designed to look at how the attentional/visual working memory load of a first target impacts the size of the SOS effect), we found mixed support for some of the predictions of the theory. Overall, searchers were more likely to miss a second lesion, but only novice searchers were more likely to miss a dissimilar lesion than a similar lesion. While experienced searchers had the same hit rates for similar- and dissimilar-second lesions, response time analyses demonstrated that both novice and experienced searchers took less time between first and second lesion detection when lesions were similar compared to when they were dissimilar. This response time analysis suggests that a first target acted as an attentional template for experienced searchers even though their second-target hit rates were not better for similar compared to dissimilar lesions. Faster response times for items similar/the same as the attentional template is a common finding in the attentional template literature.^42^

The discrepancy between experienced and novice searchers’ SOS errors for similar and dissimilar lesions was likely due to the training and experience. Regarding similar and dissimilar lesion rates, experienced searchers know that calcifications and masses can co-occur. In other words, when experienced searchers see a calcification, they may have been faster than a novice searcher to switch their attentional template to look for a mass. Novice searchers likely maintained the first target as an attentional template for longer since they are generally more unfamiliar with the lesions and had just found a direct example of a target lesion. The faster response times for experienced searchers were also likely the result of experience given that experienced searchers traditionally have to search many breast images as part of their training as a resident and at least a set number of breast images of to maintain their mammography quality standard act certification as a radiologist. Whereas novice searchers likely had slower response times because they were searching breast images for the first time.

Regarding the time frame that a first target is maintained as an attentional template, future research will need to test why and how a first target will lose its template status and how long this process may take. Given that SOS errors are known to be reduced the longer an observer searches after finding a first target,^6^ and that a first target is maintained as an attentional template, this suggests that at some point in time, a first target may not be prioritized. Research suggests that a first target may be forgotten after initial detection^22^ or transferred to long-term memory,^18^^,^^43^ which could explain why a first target loses prioritization. A third possibility, which may have occurred, is that observers may deprioritize the target naturally, which could explain why experienced searchers did not show a similarity effect while novice searchers did. For example, suppose the prioritization of the first target as the attentional template is not fruitful or searchers are trained to consistently search for many different target/lesions in an image (i.e., they have multiple attentional templates). In that case, searchers may prioritize one attentional template over another depending on the potential targets. There is some evidence that this may happen in “runs,” a known phenomenon where observers search for many targets in the same search display and consistently detect one type of target (i.e., a run) and then switch to a different type of target and consistently find the new type of target.^44^ However, it is unclear whether the switch between templates is a top-down effect (where observers consciously decide to search for a different target), a bottom-up effect (where observers happen to find a dissimilar target by chance and switch when re-primed with a different target), or both. It is reasonable to predict that observers will decide to change templates if they learn which features are likely to categorize an upcoming target.^45^ Still, it is also reasonable to predict that if observers search for longer, they are more likely to come across a target in general (whether it is a target of a similar or different type). If it is a target of a different type, this may cause them to switch their attentional template to represent the new target or possibly even maintain both targets as attentional templates of equal status given that multiple attentional templates can be held at the same time.^46^

It is clear that additional research is needed to determine if, when, and why observers deprioritize a found target as an attentional template and whether this is a top-down effect, a bottom-up effect, or both.

### 4.2 Limitations

There were key limitations to this study, and they primarily were due to using virtual breast images as opposed to real full-field digital mammograms. At the time of image generation for this study, a single DBT slice could only be generated by the Open VCT framework instead of a full-filed digital mammogram. While the use of virtual images was useful in generating matching sets of images with the only difference being whether a given lesion was present or not (see Sec. 2.3), the “realism” of these virtual images has not been compared to “real” full-field digital mammograms. While ideal observer models (i.e., computer algorithms that search with all the information pertaining to the image, including the characteristics of the lesion and lesion location and/or profile) have been used to validate these images to real images,^36^^,^^37^ future research with human searchers and real mammograms is needed to more precisely capture the true SOS rate for experienced and novice searchers. Experienced searchers may have been better than novice searchers (e.g., better hit rates, lower false alarms, and lower SOS rates) given that they read many breast images during their training/job.

A related limitation is that there was only one specific mass and one specific calcification used in the search images (i.e., the mass and calcification lesions were the same across all images) because at the time of this study, the Open VCT framework could only generate images with a specific mass and calcification. In other words, every mass and calcification used was identical and could only perceptually differ depending on the background of the image. While this was likely helpful for novice searchers (i.e., novices learned exactly what mass and calcification to look for in the practice trials), they likely would have much poorer search performance if masses and calcifications were different across images as they do not have the training to identify masses of different shapes and sizes or calcifications in different cluster arrays.

Relatedly, another potential byproduct of learning the same masses and calcification across images is that participants were less likely to retain a detected first target as an attentional template within visual working memory and maybe offloaded it to long-term memory.^18^ In other words, their attentional template prior to each search image might have been for the specific masses and calcifications used thereby facilitating the transition of a first-target attentional template to long-term memory. This process may have reduced the overall SOS effect (according to the attentional template theory) if resources that had otherwise been allocated to maintaining a first target in visual working memory were more freely available. However, the interplay between attentional templates in visual working memory and long-term memory is still a ripe area of study^47^ and our understanding of templates and their role in SOS errors is still in its infancy.^17^

Another limitation was the potential memory effects searchers may have had for the matched images. While the matched images were necessary for the SOS calculation used in this study, it is likely searchers realized that images may have repeated (even though they were not explicitly told this). To account for these limitations, future research would benefit from using: (1) virtual mammograms with a variety of different mass and calcification configurations^48^ and/or (2) a variety of real full-field digital mammograms, while testing searchers over a longer session or multiple sessions to reduce potential memory effects for matched images.

In regards to the exploratory analyses, we demonstrated how a modeling approach that leverages more of the data (i.e., trial-level data versus condition aggregates) can help deal with inter-observer variability, while simultaneously allowing us to model effects of interest (e.g., image type, false alarms). Given that substantial variation was explained by inter-observer-differences in trial response times (i.e., participant intercepts), future work may evaluate what predicts these differences, beyond experience alone (e.g., years of experience, conscientiousness, level of fatigue, time of day, etc.). The present experiment only had one experimental block (since the experiment in total was around 30 mins), so future work may also benefit from a longer experiment broken down into blocks to look at the learning function more clearly within an experiment.

Furthermore, because of the 30-min time frame, more dual-lesion images and more lesion-free, “normal” images were not included. Prior SOS research in medical image perception often utilized “matching” native-abnormality free and native-abnormality present cases to compare false positives as part of an receiver operating characteristic (ROC) calculation in determining the SOS effect.^49^ Here our methods focused on hit rates to understand the theoretical underpinnings of the SOS effect (i.e., a common way to assess SOS errors in cognitive science; see Ref. 17), and did not use ROC curves to assess the SOS effect. While it is theoretically unclear why the detection of a first target/lesion may make searchers more or less likely to make false positives^48^ future SOS research would benefit by: (1) specifically investigating the nature of false positives and (2) comparing and contrasting hit rate-SOS measures and ROC curve-SOS measures so SOS researchers utilize the appropriate SOS analyses for the questions they are trying to answer.

## 5 Conclusion

In this study, we demonstrated that SOS errors might be a significant factor influencing lesion misses in virtual mammograms and experience impacts second lesion misses after first lesion hits. The attentional template theory was used to explain why searchers were faster overall at detecting similar lesions than dissimilar lesions and why novice searchers with no breast imaging experience were more likely to miss a dissimilar lesion compared to a similar second lesion. However, it did not explain why searchers with breast imaging experience missed similar and dissimilar second lesions at the same rate. The overall hit rates did not differ between groups suggesting that differences in strategy and/or familiarity with the images likely influenced how the readers searched for the different abnormalities. Future research would benefit from using real mammograms to verify the SOS results reported here and potential differences between novice and experienced searchers not realized using virtual computer-generated mammograms.
